# Predicting the recurrence of noncoding regulatory mutations in cancer

**DOI:** 10.1186/s12859-016-1385-y

**Published:** 2016-12-03

**Authors:** Woojin Yang, Hyoeun Bang, Kiwon Jang, Min Kyung Sung, Jung Kyoon Choi

**Affiliations:** Department of Bio and Brain Engineering, KAIST, Daejeon, Republic of Korea

## Abstract

**Background:**

One of the greatest challenges in cancer genomics is to distinguish driver mutations from passenger mutations. Whereas recurrence is a hallmark of driver mutations, it is difficult to observe recurring noncoding mutations owing to a limited amount of whole-genome sequenced samples. Hence, it is required to develop a method to predict potentially recurrent mutations.

**Results:**

In this work, we developed a random forest classifier that predicts regulatory mutations that may recur based on the features of the mutations repeatedly appearing in a given cohort. With breast cancer as a model, we profiled 35 quantitative features describing genetic and epigenetic signals at the mutation site, transcription factors whose binding motif was disrupted by the mutation, and genes targeted by long-range chromatin interactions. A true set of mutations for machine learning was generated by interrogating publicly available pan-cancer genomes based on our statistical model of mutation recurrence. The performance of our random forest classifier was evaluated by cross validations. The variable importance of each feature in the classification of mutations was investigated. Our statistical recurrence model for the random forest classifier showed an area under the curve (AUC) of ~0.78 in predicting recurrent mutations. Chromatin accessibility at the mutation sites, the distance from the mutations to known cancer risk loci, and the role of the target genes in the regulatory or protein interaction network were among the most important variables.

**Conclusions:**

Our methods enable to characterize recurrent regulatory mutations using a limited number of whole-genome samples, and based on the characterization, to predict potential driver mutations whose recurrence is not found in the given samples but likely to be observed with additional samples.

**Electronic supplementary material:**

The online version of this article (doi:10.1186/s12859-016-1385-y) contains supplementary material, which is available to authorized users.

## Background

Previous cancer genome analyses were limited to protein-coding regions, which covers less than two percent of the human genome. These efforts successfully discovered a number of critical oncogenes and tumor suppressors. However, a large number of cases still remain inexplicable with those genes. The vast majority of cancer mutations are found in regions that are extraneous to protein function. These mutations in noncoding regions may act as driving factors that perturb the regulation of gene expression. The most critical criterion for identifying driving mutations is their recurrence [1]. It can be more complicated to characterize mutation recurrence in noncoding regions than in protein-coding genes. In a recent study [2], the significance of mutation recurrence at a given locus in noncoding regions was calculated by a probabilistic model based on the expected and observed mutation rates.

Recent studies suggest that the genetic and epigenetic features can determine the landscape of cancer mutations [3–6]. For example, Schuster-Böckler et al. [3] profiled a set of diverse genetic and epigenetic factors in terms of their association with the chromosomal density of cancer mutations. Polak et al. [6] found that cell-of-origin chromatin features are a strong determinant of the distribution of cancer mutations. These studies suggest that a range of genetic and epigenetic factors can be used to estimate the significance of cancer mutations. A critical aspect of regulatory mutations that has not been previously dealt with is their genes targeted by enhancer-promoter interactions. The recent growth of chromatin interactome data enables to characterize the targets of noncoding regulatory mutations. In addition, the transcription factors (TFs) whose binding is affected need to be examined, because critical noncoding mutations may act through altering the binding of tumorigenic TFs. We sought to construct a classification method that can predict functional noncoding mutations based on the significance of mutation recurrence and a wide range of related features. A total of 35 features were collected and used for the training of our classification model. Recurrence significance was assessed by testing different sizes of target and background windows.

Saturation of cancer gene discovery may be achieved with ~5000 samples per tumour type in addition to > 4700 exomes that are currently available [7]. Noncoding mutation discovery probably requires more samples because of a larger mutation target size. Millions of potential regulatory sequences exist in mammalian genomes [8]. Unfortunately, only a small number of whole-genome sequenced cancer samples have been made available. Moreover, unlike protein-changing mutations, it is complicated to identify functional noncoding variants as distinguished from inconsequential variants. In a previous work, machine learning was employed to predict noncoding mutations that exert high regulatory impacts through TF binding disruption [9]. These prompted us to develop a machine learning method that can learn the features of the mutations that are recurrent in a small-sized cancer cohort and then predict functional mutations whose recurrence may appear with additional samples.

## Results

### Study overview

**Fig. 1:**
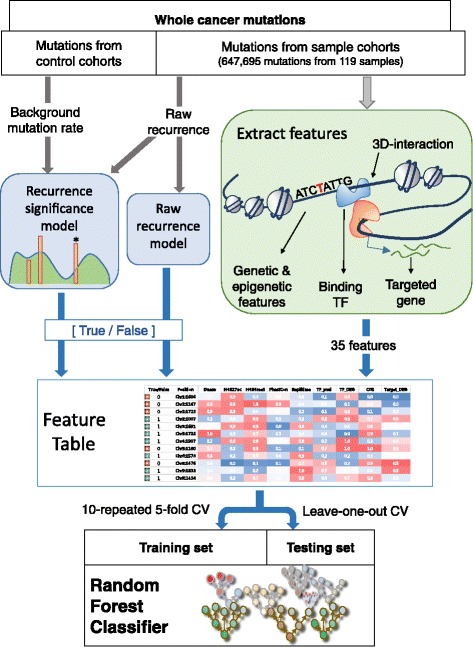
The overall schematic workflow. The construction of the training set and the training of our random forest classifier that aims to predict recurring mutations in breast cancer

The overall schematic workflow of this study is depicted in Fig. 1. We obtained whole-genome pan-cancer somatic mutation calls from public resources and used breast cancer somatic mutations to obtain the instances of the training set for the classifier. We examined the recurrence of each mutation among the pan-cancer samples and also measured the statistical significance of the recurrence level. The raw count or statistical significance of recurrence was used to classify the mutations into true and false sets. Of the breast cancer mutations, we investigated those in potential regulatory regions of protein-coding genes through enhancer-promoter interactions. We profiled the genetic and epigenetic features of the mutation sites and the cancer-related or functional features of the target genes mapped to the mutations. In addition, we examined the features of the TFs whose binding sites were expected to undergo motif gain or loss due to the mutations.

Based on these mutation-, gene-, and TF-related features, we trained a random forest classifier to predict recurring mutations. We performed a 10-repeated 5-fold cross validation (CV) and used the receiver operating characteristic (ROC) curve to quantify the classification performance. Random forest was employed because the ensemble method usually shows higher performance in multi-dimensional problem space as compared to conventional methods such as support vector machine (SVM). We compared the performance of SVM and artificial neural network (ANN) with that of random forest (Additional file 1: Figure S1). In addition, we conducted a leave-one-out-cross-validation (LOOCV). Through the LOOCV, we predicted important mutations from a test set including instances of only one sample and a training set consisting of instances from the rest of the samples.

### Performance evaluation of raw and significant recurrence models

We trained a random forest classifier for a total of 24 sets of true and false mutations based on the raw recurrence and recurrence significance model. The first 12 sets were based on the raw recurrence model. Raw recurrence was calculated in the window of 5 bp, 10 bp, or 20 bp, and the mutation was assigned to the true set if the recurrence count was greater than a cutoff (≥2, ≥ 3, ≥ 4, or ≥ 5) (Table 1). The other sets were generated based on the recurrence significance model. Recurrence was first obtained in the window of 5 bp, 10 bp, or 20 bp, and then its statistical significance was computed based on the background window of 1 kbp, 10 kbp, 100 kbp, or 1 Mbp (Table 2). The mutation was assigned to the true or false set with the *p*-value cutoff of 5 × 10^−6^ for the recurrence significance model. The false sets were randomly composed as a subset of non-recurrent mutations such that they were three times the size of the true sets.

**Table 1:** Performance of recurrent mutation prediction: Raw recurrence model

Recurrence window	Raw recurrence threshold	Size of true set	Average AUC	Randomized AUC
20 bp	≥2	4473	0.57	0.50
	≥3	866	0.64	0.50
	≥4	216	0.73	0.51
	≥5	94	0.74	0.47
10 bp	≥2	2795	0.58	0.50
	≥3	466	0.67	0.50
	≥4	114	0.75	0.50
	≥5	43	0.77	0.52
5 bp	≥2	1893	0.61	0.52
	≥3	292	0.68	0.47
	≥4	61	0.78	0.58
	≥5	21	0.65	0.51

**Table 2:** Performance of recurrent mutation prediction: Significant recurrence model

Recurrence window	Background window	Size of true set	Average AUC	Randomized AUC
20 bp	1Mbp	210	0.71	0.51
	100kbp	212	0.71	0.50
	10kbp	238	0.70	0.53
	1kbp	191	0.70	0.51
10 bp	1Mbp	114	0.78	0.55
	100kbp	114	0.78	0.51
	10kbp	133	0.76	0.46
	1kbp	99	0.76	0.49
5 bp	1Mbp	201	0.72	0.48
	100kbp	198	0.72	0.47
	10kbp	181	0.72	0.47
	1kbp	131	0.73	0.43

The sets of true and false mutations were cross-validated using the random forest classifier based on 35 features (Additional file 2: Table S1). Prediction accuracy was calculated by averaging 10 5-fold CVs. Then, we drew an ROC curve of prediction accuracy and quantified the performance of the classifier using the AUC (Tables 1 and 2). The AUC values of the 12 test sets from the raw recurrence model (Table 1) indicate that our classifiers were trained properly when the raw recurrence count was 4 or higher. In particular, the recurrence window of 10 bp generally outperformed 5 bp or 20 bp. We considered the statistical significance of recurrence to account for local background mutation frequency. First, in order to compare the significance model with the raw recurrence method, the *p*-value threshold was adjusted to 5 × 10^−6^ to render the number of mutations in the true set comparable to the size of the true set in the raw recurrence model (100 ~ 200 mutations for raw recurrence ≥ 4). We performed 10-repeated 5-fold CV for varying window size (Table 2). As a result, we determined that the 10 bp window was most suitable for the significance model. For the 10 bp window, the highest AUC was achieved when the background window was 1 Mbp or 100 kbp. However, because smaller true sets generally resulted in better AUCs, we further adjusted the *p*-value cutoff, and found that the 10 kbp and 100 kbp background windows performed better than the 1 Mbp window when the size of the true set was comparable (Additional file 1: Figures S2–S3). Therefore, the 10 kbp and 100 kbp windows were considered as the best background size for the recurrence significance model. Meanwhile, our classifiers trained using randomized true/false labels showed the average AUC of 0.43 ~ 0.58 (Tables 1 and 2).

### Comparison of raw and significant recurrence model

**Fig. 2:**
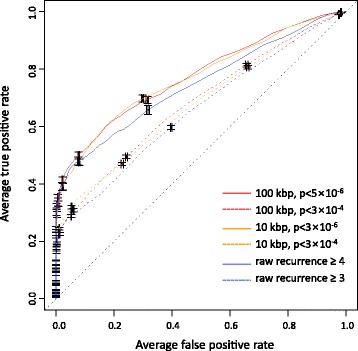
ROC curves comparing the raw recurrence and significant recurrence model

As seen in Tables 1 and 2 and (Additional file 1: Figure S4), the AUC of the recurrence significance model was not explicitly superior to that of the raw recurrence model. We speculated that the number of mutations in the training set mattered because the AUC from smaller true sets tended to be higher. In order to equalize the numbers of true mutations in the training sets, we adjusted the *p*-value of the significance model. As a result, for the significance model of 10 bp windows, the *p*-value thresholds were set to 3 × 10^−6^ for 10 kbp and 5 × 10^−6^ for 100 kbp background windows. With these thresholds, training sets with background windows of 10 kbp and 100 kbp both resulted in 114 true mutations, which was the same as ≥ 4 raw recurrence counts in 10 bp windows (Table 1). Similarly, to match the significant recurrence model with the raw recurrence model of ≥3 counts, we used the additional *p*-value cutoff of 3 × 10^−4^. With this threshold, the numbers of the true mutations that were filtered using 10 kbp and 100 kbp background windows were 474 and 452, which were close to 466 for ≥3 recurrence counts in the raw recurrence model. Using the adjusted *p*-value cutoffs, we compared the raw recurrence model of 10 bp windows with two significance models of 10 bp windows (10 kbp and 100 kbp background windows) based on the ROC curve (Fig. 2). As a result, the classifier showing the highest performance was from the significance model with the 10 bp recurrence window, 100 kbp background window, and *p*-value cutoff of 5 × 10^−6^ (red solid line in Fig. 2). This particular classifier showed an AUC of 0.78.

### Prediction on new or external samples

**Fig. 3:**
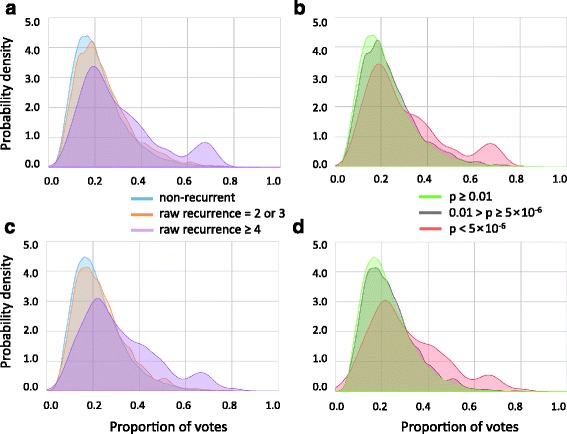
Proportion of positive votes by 1000 decision trees of the random forest. **a**-**b** Proportion of votes by the classifier trained with the raw recurrence model (≥4) for mutations in the test samples. **c**-**d** Proportion of votes by the classifier trained with the significant recurrence model (*p*-value < 5 × 10^−6^) for mutations in the test sample. **a**, **c** Voting for mutations having no (0), moderate (2 or 3) recurrence, or high (≥4) raw recurrence. **b**, **d** Voting for mutations whose recurrence significance was low (p ≥ 0.01), moderate (5 × 10^−6^ ≤ *p* < 0.01), or high (*p* < 5 × 10^−6^)

We employed an LOOCV to test the utility of our classifier in predicting significant recurrent mutations in a new cancer sample based on the features learned from reference cohort samples. Our classifier was trained by using mutations in 118 breast cancer samples and then used to predict significant mutations in the one sample left out from the training process. We observed the proportion of the mutations that are truly recurrent according to the significance of the prediction result for each mutation as measured by the number of positive votes by 1000 decision trees in our random forest classifier (Fig. 3). By a majority vote of the individual trees, both the classifier trained with the raw recurrence model (Fig. 3a-b) and the classifier trained with the significant recurrence model (Fig. 3c-d) preferentially identified mutations that were actually recurrent in terms of the raw recurrence count (Fig. 3a and c) or the recurrence *p*-value (Fig. 3b and d).

**Fig. 4:**
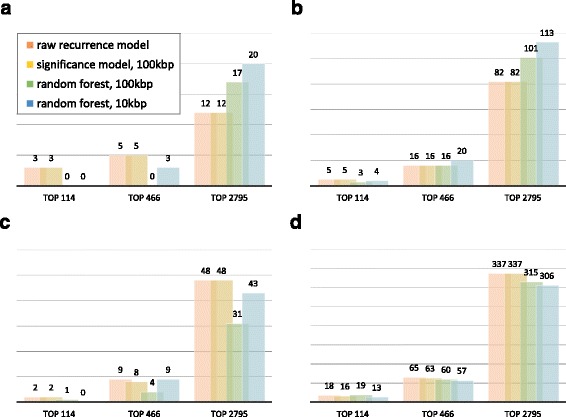
Number of mutations whose recurrence was revealed by sample addition. The top-voted mutations from the random forest trained with the significant recurrence model (100 kbp or 10 kbp as the background window) were compared to the same number of the most highly recurrent mutations based on the raw or significance model. **a**-**b** Recurrence recalculated in the window of (**a**) 10 bp and (**b**) 100 bp after the addition of new breast cancer samples. **c**-**d** Recurrence recalculated in the window of (**c**) 10 bp and (**d**) 100 bp after the addition of lung cancer samples

There were mutations that were actually recurrent but not positively predicted. These may indicate inconsequential mutations that do not share the features of functional regulatory mutations. On the contrary, there were mutations that were positively predicted but not actually recurrent (the right tails of the curves for the non-recurrent mutations in Fig. 3). There is a possibility that the recurrence of these mutations is not detected because of limited sample size. To estimate the capability of our method to predict mutations whose recurrence can be revealed only by additional samples, we recalculated the recurrence of each mutation using external datasets which were not used when training our classifier. We observed that the top-voted mutations from our random forest classifier were more likely to exhibit their recurrence upon sample addition than most recurrent mutations from the raw recurrence model or most significant mutations from the significance model (Fig. 4a and b). However, this pattern was not observed when we used lung cancer instead of breast cancer as external samples (Fig. 4c and d), in agreement with the fact that our random forest predictor relied on the features of the genome, epigenome and transcriptome of breast cancer. In addition, the random forest classifier trained based on the significance model with the background window size of 10 kbp outperformed that based on the background window of 100 kbp.

### Measuring variable importance

Variable importance was measured by the Mean Decrease Accuracy of random forest (Table 3). Among others, the chromatin accessibility signals (DnaseSig) at the mutated site in the cancer cells and cells-of-origin stood out as the most important feature in both the raw recurrence and significant recurrence model. Other important features with high importance in both tests included the cancer gene score (CGS) of target genes in the protein interactome (InteractNet.CGS_L1 and InteractNet.CGS_L2), the sum of the differentially gene expression scores between cancer and normal for the downstream genes of the mutation target genes in the regulatory network (RegNet.DEG_score), the distance between the mutation site and its nearest cancer risk locus (Distance.to.GWAS), the H3K27me3 signals in the cancer cells, and the H3K4me1 signals in the cells-of-origin.

**Table 3:** Feature list and variable importance

Category		Mean decrease accuracy (%)	
		*p* < 5 × 10^−6^	Recurrence ≥ 4
Target Gene Features	HumanNet.CGS_L1	5.26	4.85
	HumanNet.CGS_L2	6.78	6.42
	InteractNet.CGS_L1	10.86	9.52
	InteractNet.CGS_L2	10.16	10.84
	RegNet.DEG_score	10.88	10.79
	DEG_score	9.72	9.51
Binding TF Features	HumanNet.CGS_L1	4.91	2.92
	HumanNet.CGS_L2	4.57	3.23
	InteractNet.CGS_L1	2.67	2.66
	InteractNet.CGS_L2	4.99	7.40
	RegNet.DEG_score	6.68	3.61
	DEG_score	4.77	6.13
TFBS Scores	diff_Log_Pval_FIMO	2.59	2.53
	avg_Log_Pval_FIMO	8.04	7.48
	Gain_or_Loss	1.57	0.37
Cancer Cell Epigenetic Signals	DnaseSig	17.34	16.94
	H3K27ac	8.95	7.62
	H3K27me3	11.94	10.23
	H3K36me3	7.50	5.23
	H3K4me3	9.45	7.99
	H3K9me3	10.19	9.02
Genetic Signals	Distance.to.GWAS	15.82	11.00
	Early.to.late_Rate	7.09	7.00
	PhastCons	1.26	−0.18
Original Cell Epigenetic Signals	DnaseSig	17.90	17.05
	H3K27ac	9.45	9.59
	H3K27me3	5.91	7.26
	H3K36me3	15.69	7.98
	H3K4me1	11.50	9.69
	H3K4me2	7.97	8.78
	H3K4me3	7.88	7.02
	H3K79me2	6.33	4.98
	H3K9ac	7.79	8.79
	H3K9me3	4.83	13.12
	H4K20me1	8.38	7.44

Meanwhile, the H3K9me3 and H3K36me3 signals in the cells-of-origin but not in the cancer cells stood out under both recurrence models. In addition, the importance measure for the H3K9me3 signals in the raw recurrence model significantly differed from the significance model. This may reflect the fact that heterochromatin has high mutation rates due to the lack of DNA damage repair activity. In contrast, the importance of H3K36me3 in the significance model was remarkably higher than that in the raw recurrence model. Biological implications of each variable’s importance remain to be further investigated.

## Conclusions

We applied machine-learning techniques to facilitate the discovery of important mutations in cancer. Our machine leaning method was capable of processing multiple features concertedly, enabling us to consider various genetic and epigenetic factors that have direct or indirect relevance to mutagenesis and tumorigenesis. Of particular importance and novelty, one of the factors that turned out to be critical in the prediction processes was the oncogenic relevance of mutation target genes. This factor was made available by leveraging the data of three-dimensional chromatin structure that provide long-range interactions between promoters and enhancers. We considered the expression change of the genes due to the linked mutation, their effects on downstream genes in the regulatory network, and their physical or functional interactions with known cancer genes. An additional benefit of using the chromatin interactome data was that we were able to filter mutations residing in regulatory regions.

We observed that both the raw and significant recurrence models worked properly. We found a proper window size for recurrence identification to be 10 bp, with which our classifier achieved the highest performance under the significance model. Meanwhile, the background mutation rates calculated within a 100 kbp window showed the best performance. In addition, we proposed important features to consider when inferring cancer-driving mutations. For example, the chromatin accessibility signals showed particularly high explanatory power. We also identified other features such as the distance of the mutation site to known cancer risk loci and several histone marks that signify regulatory activity. As described above, our network-based scores for target genes also played an important role in mutation classification. Overall, most of the identified features had biological relevance.

Our method was able to find putative driver mutations that show low or no recurrence in currently available samples but may show recurrence when additional cancer samples are interrogated. Our analyses using external additional samples propose that our machine learning approach will make it possible to expand the catalog of putative noncoding driver mutations until mutation discoveries have reached saturation through large-scale whole-genome analyses.

## Methods

### Dataset for mutation candidates

We obtained public whole-genome mutation call data for 507 samples across 10 different cancer types [10] and 2297 samples across 18 cancer types from The Cancer Genome Atlas (TCGA) data portal. In order to eliminate any residual germ line mutations, all samples were filtered by the 1000 genome variants [11]. We chose 119 breast cancer sample with 647,695 somatic mutations for the learning and evaluation of our classifier.

### Target gene mapping

To map noncoding mutations to their target genes, we employed two datasets for enhancer-promoter pairs. First, we connected the mutations to the genes by using the integrated method for predicting enhancer targets (IM-PET), which was used to predict putative EP pairs across 12 cell types by characterizing multiple genomic features [12]. For the mutations of breast cancer, we used IM-PET for the MCF-7 cell line. Second, the mutations not connected via the IM-PET were mapped to their target genes by the DNase I hypersensitive site (DHS) correlation map [13]. The DHS correlation map was constructed by computing DHS tag density correlations across 349 diverse cell types between all possible proximal and distal DHS pairs within 500 kb. The mutations were mapped to the gene that had the maximum DHS correlation across all cell types.

### TF mapping

The position weight matrices for TFs were downloaded from the TRANSFAC database [14] available at http://www.gene-regulation.com/. The TF binding motifs within DHS regions were identified using the FIMO program [15]. FIMO was run for the wild-type and mutated sequence separately. Motif gain or loss was identified when only the mutant and wild-type exceeded the *p*-value threshold, respectively. When motif gain or loss was identified, the binding gene scores (Table 3) were obtained for the corresponding TF. The diff_Log_Pval_FIMO binding score was defined as the logarithmic difference in the FIMO *p*-value between the wild-type and mutant sequences, the avg_Log_Pval_FIMO was calculated by averaging minus log of the two *p*-values, and the Gain_or_Loss was set to 1 (gain) or −1 (loss). On the contrary, when there was no motif change due to the mutation, the most likely binding site (lowest FIMO *p*-value) was identified within the entire DHS region containing the mutation. In this case, the diff_Log_Pval_FIMO was set to 0, the avg_Log_Pval_FIMO to the minus log of the lowest FIMO *p*-value, and the Gain_or_Loss to 0 (indicating no change). The DHS regions were obtained from the ENCODE’s uniformly processed DNase-seq peaks in the MCF-7 cell line [16].

### Gene expression change features

We computed the Differentially Expressed Gene (DEG) score by quantifying the difference of gene expression between cancer and normal samples as a learning feature of our random forest. For this, we obtained RNA sequencing data for tumor and matched normal samples from the TCGA data portal. The expression values were assumed to follow a normal distribution. The *p*-value was calculated by the Students’ t-test based on the hypothesis that there was no difference between cancer and normal. We defined the DEG score as the negative common logarithm of the *p*-value as below:

$$ \mathrm{D}\mathrm{E}\mathrm{G}\_\mathrm{score}=\frac{x}{x+1},\;\mathrm{where}\;x=- \log\;\left(p\hbox{-} \mathrm{value}\;\mathrm{of}\;\mathrm{t}\hbox{-} \mathrm{test}\right) $$

In addition, RegNet.DEG_score was defined as the sum of the expression changes of all genes regulated by the target gene or TF mapped to the mutation. Here, we used a previously constructed Bayesian regulatory network in breast cancer [17]. We summed the DEG_score of all downstream genes reachable within 3 hops from the mutation target gene or TF in the regulatory network.

### Network features

We also used the network-related features of the mutation target gene and binding TF. Specifically, the cancer gene score (CGS) was defined to measure the degree of connections with tumor suppressors or oncogenes [1, 18] in the following networks: (1) an integrated physical interaction network (Interactome), created by merging the yeast-two-hybrid proteome-scale interactions [19], literature-based protein-protein interactions [19], binary interactions identified from Stitch-seq [20], and high-quality protein interactome from the HINT database [21], and (2) HumanNet [22], a probabilistic functional network of human genes, constructed by a modified Bayesian integration of 21 types of omics data from multiple organisms. InteractNet.CGS and HumanNet.CGS were defined as the number of known cancer genes interacting with the mutation target gene in the Interactome and HumanNet. Additional suffixes _L1 and _L2 indicate the hop count 1 and 2 from the target gene to the cancer gene, respectively.

### Genetic signal features

For each mutation site, we considered three genetic features: the distance to cancer risk loci, replication timing, and evolutionary conservation score. To measure the degree of relation with cancer risk loci, we calculated the distance to the nearest cancer-related genome-wide association study (GWAS) SNPs from each mutation position. We obtained a total of 1002 SNPs for 45 different tumour types from the GWAS catalogue [23]. The replication timing was calculated using data from the study conducted by Hansen et al. [24]. Raw reads were downloaded for four cell-cycle fractions (G1B, S1, S4 and G2), averaged to 4 million reads each, and normalized to percentage replication per nucleotide position. An early-to-late ratio was calculated as (G1B + S1)/(S4 + G2). We computed a conservation score at each mutation position using phastCons [25], available at the UCSC GoldenPath genome resource, which assigned a conservation score based on a phylogenetic hidden Markov model for the estimation of the probability that each nucleotide belongs to a conserved element.

### Epigenetic signal features

We collected several epigenetic signal features for the MCF-7 cell line from the ENCODE project [16]: DNase-seq signals and ChIP-seq signals for several important histone marks – H3K27ac, H3K27me3, H3K36me3, H3K4me3, and H3K9me3. We also collected epigenetic data of the human normal breast cell line, HMEC, including DNase-seq signals and ChIP-seq signals for histone marks such as H3K27ac, H3K27me3, H3K36me3, H3K4me1, H3K4me2, H3K4me3, H3K79me2, H3K9ac, H3K9me3, and H4K20me1.

### Raw and significant recurrence models

**Fig. 5:**
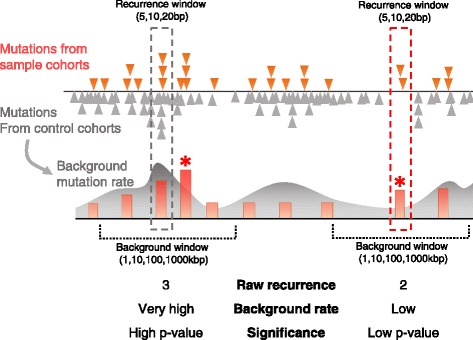
Illustration of the raw recurrence and significant recurrence model. (*Left dotted box*) Raw recurrence represented as the orange bar is not significant relative to the expected occurrence (*green curve*) as estimated from the background mutation rates. (*Right dotted box*) On the contrary, mutation recurrence (*orange bar with asterisk*) is statistically significant considering a low probability of background mutation occurrence

In the raw recurrence model, we mapped the pan-cancer mutations in windows of various sizes (5 bp, 10 bp, and 20 bp) centered on the breast cancer mutations. The number of overlapping mutations within a window was used as the raw recurrence count. Meanwhile, in the significance model, the statistical significance of the mutation of interest was estimated based on the background mutation rate in 1 kbp to 1 Mbp windows (Fig. 5). We assumed that mutation density follows the Poisson distribution. Therefore, the Poisson variable estimates the level of mutation occurrence expected when there is no biological effect (i.e., when there is no recurrence). Mutation occurrence is expected to increase in proportion to the window size and background mutation rate. Therefore, when *w* the size of the recurrence window and *p* the background mutation rate per single base pair, the approximate *p*-value was calculated as below:

$$ P\ \mathrm{value}\ \left(\mathrm{recurrence}\ \mathrm{of}\ \mathrm{site}\ \mathrm{is}\ x\right)=\mathrm{P}\left(X\ \ge x\right),\ \mathrm{where}\ X \sim \mathrm{Poisson}(wp). $$

In other words, the given recurrence level, *x*, is tested against the Poisson distribution, from which the estimated recurrence level, *X*, is drawn. We computed the *p*-values for all recurrent mutations using an R package.

### Random forest classifier and cross-validation

Mutations were classified into true or false training sets based on their raw recurrence or statistical significance as described in the previous session. In the training dataset, the true sets were comprised of highly recurrent or significant mutations and the false sets were composed as random sets of non-recurrent mutations. The false sets were three times the size of the true sets. Then, we trained a random forest classifier consisting of 1000 decision trees based on the features described above. We used the algorithm implemented in the R package randomForest (http://www.r-project.org/) [26, 27]. The classification performance was evaluated by a 10-repeated 5-fold cross validation. We trained classifiers using various sets of true and false mutations differing by the recurrence window size (5 bp, 10 bp, and 20 bp) and the statistical background window size (1 kbp, 10 kbp, 100 kbp, and 1 Mbp). The trained models were then evaluated by the AUC of the ROC curve. We then selected classifiers showing high performance, and performed a further validation by LOOCV with the selected parameters. The LOOCV method used all the mutations from one sample as the testing set and the mutations from the remaining samples as the training set. The training set was created and processed in the same manner as 5-fold cross validation. We observed the proportion of votes of each mutation in the test set and analyzed with raw recurrence and recurrence significance. In addition, the importance of all variables in each classifier was measured as the Mean Decrease Accuracy defined in the randomForest R package. Specifically, the importance of the *n*th variable was estimated by randomly permuting all the values of the *n*th variable in the training set for each classifier. Decreased accuracy correlates with the importance of the given variable. The variable of high importance could be considered as a feature that many significant mutations share.

### Validation using an external dataset

In order to estimate the prediction performance of our random forest, we recalculated the recurrence of the positively predicted mutations after adding whole-genome cancer samples that were not included in the training. Specifically, for the top-voted mutations in our random forest prediction, we checked whether there are additional mutations in the new samples within the recurrence window. Recurrent mutations were selected by their raw recurrence, recurrence significance, and proportion of votes by two random forest classifiers built with the LOOCV. The two classifiers were built based on the mutations from the significance model with the 10 bp window, *p*-value cutoff of 5 × 10^−6^, and background window of two different sizes (10 kbp and 100 kbp). Using the raw recurrence model, we selected 114, 466, and 2795 recurrent mutations with the threshold counts of ≥ 4, ≥ 3, and ≥ 2, respectively. Then, we matched them with the same numbers of most significant mutations from the significance model and top-voted mutations from the random forest classifiers (Fig. 5). Recurrence levels were recalculated with new breast or lung cancer samples by examining the numbers of mutations in the window of 10 bp or 100 bp centered on the mutation of interest. Variant calls for the external cancer samples, composed of 92 breast and 90 lung cancer cases, were downloaded from the TCGA portal.

## Additional files

Additional file 1: Figure S1. Performance comparison between random forest (RF), support vector machine (SVM) and artificial neural network (ANN). **Figure S2** Performance comparison for various sizes of recurrence window and background window. **Figure S3** Performance for the combinations of recurrence and background window sizes. **Figure S4** Comparison of the raw recurrence model and significant recurrence model. (PDF 771 kb)
Additional file 2: Table S1. Annotation of random forest input features. (XLSX 13 kb)

## Abbreviations

AUC: Area under the curve
bp: Base pair
CGS: Cancer gene score
CV: Cross validation
DEG: Differentially Expressed Gene
DHS: DNase hypersensitive site
GWAS: Genome-wide association study
IM-PET: Integrated methods for predicting enhancer targets
LOOCV: Leave-one-out-cross-validation
ROC: Receiver operating characteristic
TF: Transcription factor
TFBS: Transcription factor binding sites
