# Supplementary material for: Predicting the recurrence of noncoding regulatory mutations in cancer
Source: BMC Bioinformatics. 2016 Dec 3;17:492. doi: 10.1186/s12859-016-1385-y (PMC5135808; doi:10.1186/s12859-016-1385-y)
Supplement: Additional file 1: Figure S1. — Performance comparison between random forest (RF), support vector machine (SVM) and artificial neural network (ANN). Figure S2 Performance comparison for various sizes of recurrence window and background window. Figure S3 Performance for the combinations of recurrence and background window sizes. Figure S4 Comparison of the raw recurrence model and significant recurrence model. (PDF 771 kb) [file 12859_2016_1385_MOESM1_ESM.pdf]

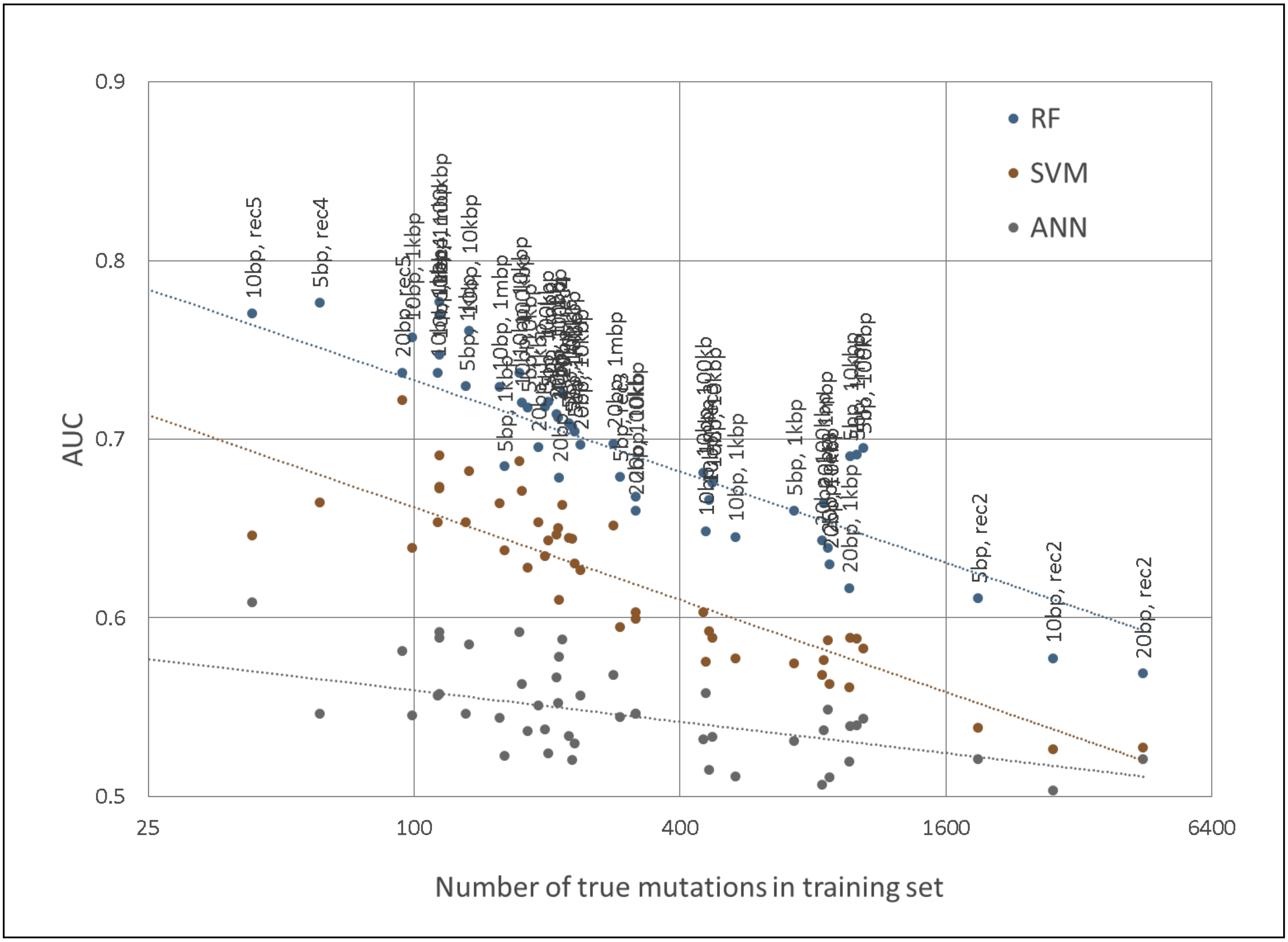

Figure S1. Performance of classification comparing 3 kinds of machine learning method - random forest (RF), support vector machine (SVM) and artificial neural network (ANN). Performance is quantified as AUC of ROC. Cross-validation is based on 5-fold cross-validation (5FCV) and AUC values are averaging 10-repeated 5FCV. Blue line represents logarithmic trend line and indicates RF method over-performs SVM (orange line) and ANN (grey line). AUC of RF is about 0.05 higher than SVM If they share same training sets. ANN shows least AUC and more different from other methods when set of mutations are small. We suppose this is because performance of ANN is influenced by over-fitting.

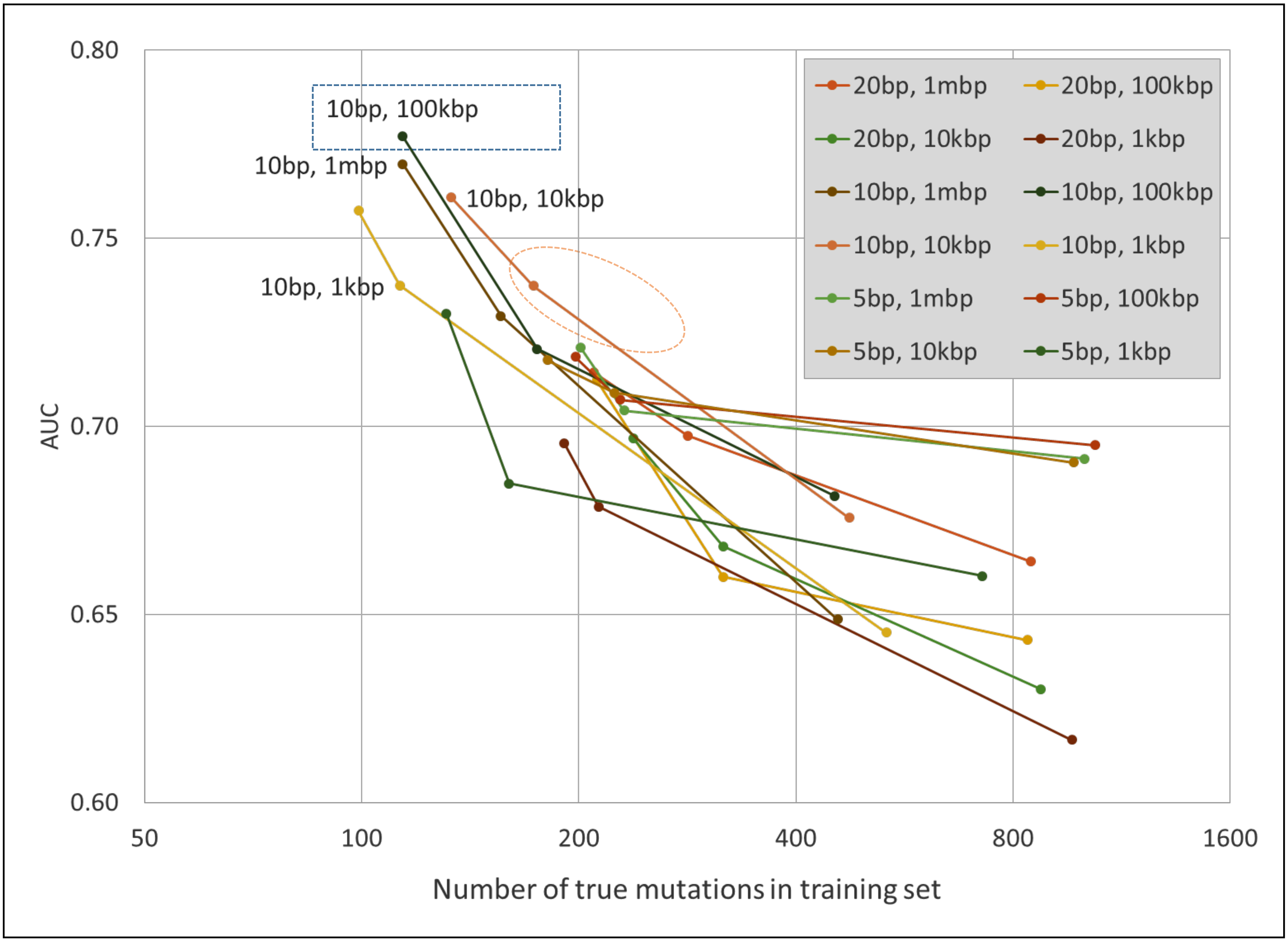

Figure S2. Performance of classification comparing various recurrence window and background window.

With recurrence significance model, random forest (RF) shows highest AUC of ROC when recurrence window is 10 bp and background window is 100 kbp (blue rectangle). This condition shows AUC of 0.78 with p-value of 5×10^{-6} as cutoff value for true mutation. Case of 10 kbp as background window is also distinguishing. AUC is lower than 100 kb in best condition, but higher when the size of true mutation set is over 150 (orange oval). For the reason, we decided to test 10 kbp and 100 kbp with in-depth analysis.

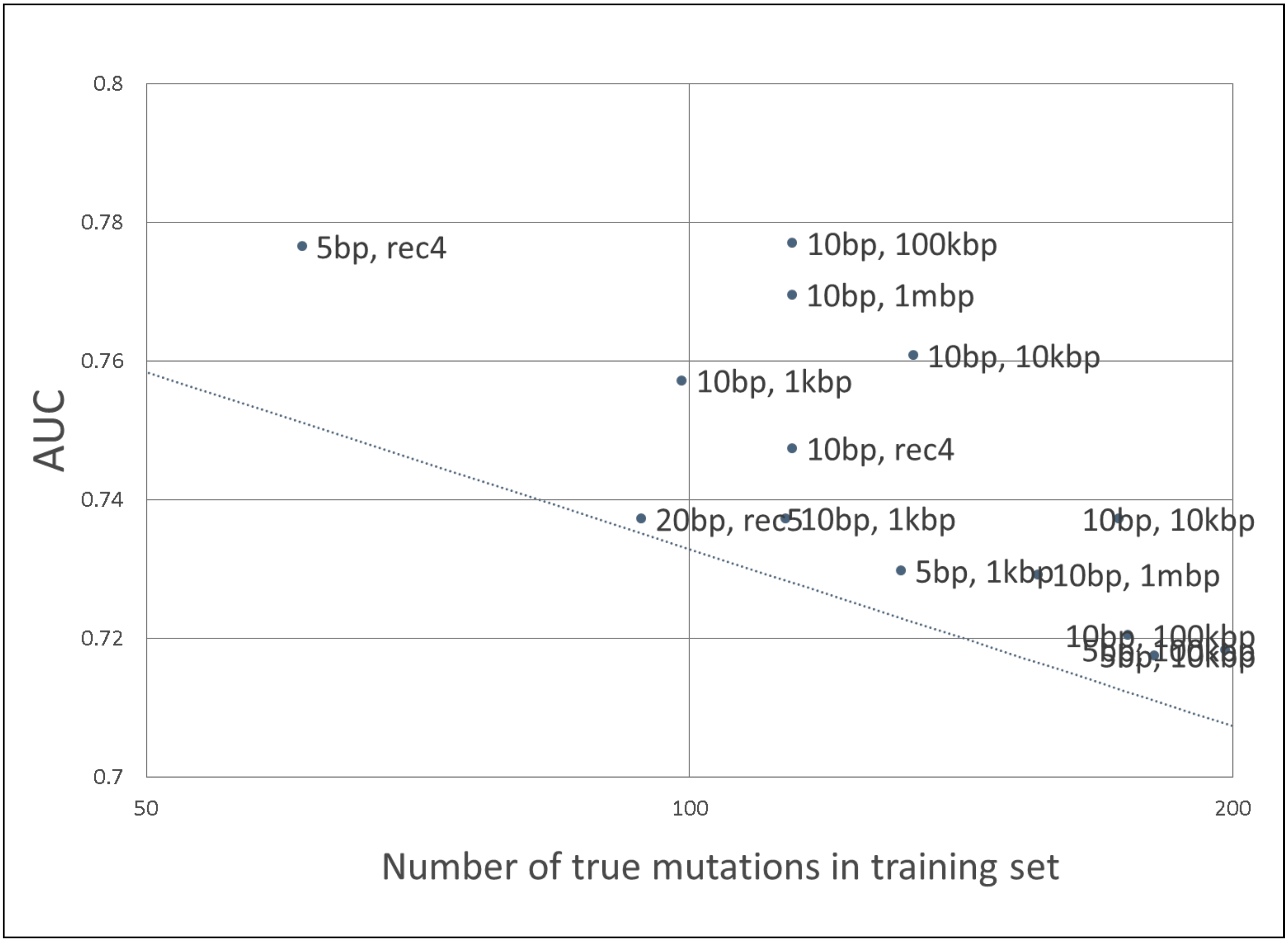

Figure S3. Magnification of figure S1 to find out best performance of classification. Random forest (RF) shows highest AUC when recurrence window is 10 bp and background window is 100 kbp with recurrence significance model. With 10 bp and 100 kbp as recurrence and background window size, the AUC is about 0.78 when p-value cutline is 5×10^{-6} and size of true mutation set is 114. With same size of true set, RF shows AUC of 0.75 from raw recurrence model with window size of 10 bp.

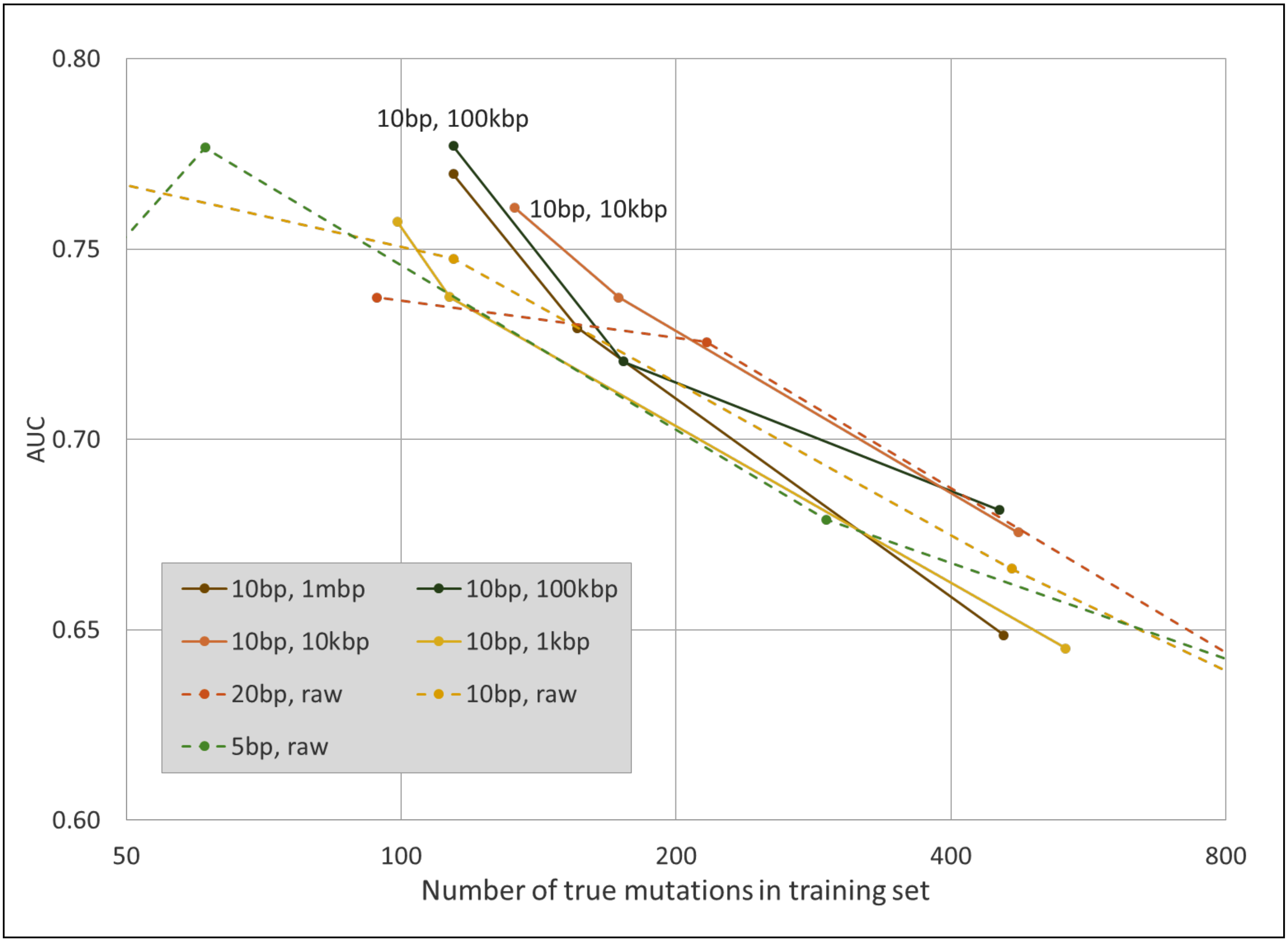

Figure S4. Comparison of raw recurrence model and significant recurrence model. Random forest (RF) shows slightly better performance with the significant recurrence model (solid lines) than the raw recurrence model (dotted lines). Best condition for RF is when the number of true mutations is around 110. In that case, the significant recurrence model shows 0.02 higher AUC than raw recurrence model.
